# Biomedical consequences of elevated cholesterol-containing lipoproteins and apolipoproteins on cardiovascular and non-cardiovascular outcomes

**DOI:** 10.1038/s43856-022-00234-0

**Published:** 2023-01-20

**Authors:** Amand F. Schmidt, Roshni Joshi, Maria Gordillo-Marañón, Fotios Drenos, Pimphen Charoen, Claudia Giambartolomei, Joshua C. Bis, Tom R. Gaunt, Alun D. Hughes, Deborah A. Lawlor, Andrew Wong, Jackie F. Price, Nishi Chaturvedi, Goya Wannamethee, Nora Franceschini, Mika Kivimaki, Aroon D. Hingorani, Chris Finan

**Affiliations:** 1 Institute of Cardiovascular Science, Faculty of Population Health, University College London, London, UK; 2 UCL BHF Research Accelerator Centre, London, UK; 3 Department of Cardiology, Division Heart and Lungs, University Medical Center Utrecht, Utrecht University, Utrecht, The Netherlands; 4 Department of Cardiology, Amsterdam Cardiovascular Sciences, Amsterdam University Medical Centre, University of Amsterdam, Amsterdam, The Netherlands; 5 Department of Life Sciences, College of Health, Medicine and Life Sciences, Brunel University London, Uxbridge, UK; 6 Department of Tropical Hygiene, Faculty of Tropical Medicine, Mahidol University, Bangkok, 10400 Thailand; 7 Integrative Computational BioScience (ICBS) Center, Mahidol University, Bangkok, 10400 Thailand; 8 Istituto Italiano di Tecnologia, Non-coding RNAs and RNA-based Therapeutics, Via Morego, 30, 16163 Genova, Italy; 9 Cardiovascular Health Research Unit, Department of Medicine, University of Washington, Seattle, WA USA; 10 MRC Integrative Epidemiology Unit, Bristol Medical School, University of Bristol, Bristol, BS8 2BN UK; 11 NIHR Bristol Biomedical Research Centre, University Hospitals Bristol National Health Service Foundation Trust and University of Bristol, Bristol, UK; 12 MRC Unit for Lifelong Health and Ageing, University College London, London, UK; 13 Population Health, Bristol Medical School, University of Bristol, Bristol, UK; 14 Usher Institute, University of Edinburgh, Edinburgh, UK; 15 Primary Care and Population Health, University College London, London, UK; 16 Department of Epidemiology, Gillings School of Global Public Health, University of North Carolina, Chapel Hill, NC USA; 17 Department of Mental Health of Older People, Division of Brain Sciences, University College London, London, UK

**Keywords:** Lipids, Biomarkers, Epidemiology, Genome-wide association studies

## Abstract

**Background:**

Higher concentrations of cholesterol-containing low-density lipoprotein (LDL-C) increase the risk of cardiovascular disease (CVD). The association of LDL-C with non-CVD traits remains unclear, as are the possible independent contributions of other cholesterol-containing lipoproteins and apolipoproteins.

**Methods:**

Nuclear magnetic resonance spectroscopy was used to measure the cholesterol content of high density (HDL-C), very low-density (VLDL-C), intermediate-density (IDL-C), as well as low-density lipoprotein fractions, the apolipoproteins Apo-A1 and Apo-B, as well as total triglycerides (TG), remnant-cholesterol (Rem-Chol) and total cholesterol (TC). The causal effects of these exposures were assessed against 33 outcomes using univariable and multivariable Mendelian randomization (MR).

**Results:**

The majority of cholesterol containing lipoproteins and apolipoproteins affect coronary heart disease (CHD), carotid intima-media thickness, carotid plaque, C-reactive protein (CRP) and blood pressure. Multivariable MR indicated that many of these effects act independently of HDL-C, LDL-C and TG, the most frequently measured lipid fractions. Higher concentrations of TG, VLDL-C, Rem-Chol and Apo-B increased heart failure (HF) risk; often independently of LDL-C, HDL-C or TG. Finally, a subset of these exposures associated with non-CVD traits such as Alzheimer’s disease (AD: HDL-C, LDL-C, IDL-C, Apo-B), type 2 diabetes (T2DM: VLDL-C, IDL-C, LDL-C), and inflammatory bowel disease (IBD: LDL-C, IDL-C).

**Conclusions:**

The cholesterol content of a wide range of lipoprotein and apolipoproteins associate with measures of atherosclerosis, blood pressure, CRP, and CHD, with a subset affecting HF, T2DM, AD and IBD risk. Many of the observed effects appear to act independently of LDL-C, HDL-C, and TG, supporting the targeting of lipid fractions beyond LDL-C for disease prevention.

## Plain language summary

It is known that increases in the amount of certain fats and proteins in the blood can lead to heart attacks. These increases are also found in people with other diseases. Here, we looked at inherited differences in some fats and proteins in blood to explore whether these could be associated with various diseases. We found that some fats and proteins in blood were associated with heart disease (including heart failure), blood pressure, blockages in blood vessels, and to a lesser extent with diabetes, Alzheimer’s disease, and inflammatory bowel disease. These findings suggest that changes to lipids and proteins in the blood might lead to various diseases, including some that are not normally associated with changes in the blood. Monitoring these changes could improve diagnosis and treatment of these diseases.

## Abstract (web summary)

Schmidt et al. evaluate the effects of elevated circulating concentrations of cholesterol-containing lipoproteins and apolipoproteins. Effects are seen on measures of atherosclerosis, blood pressure, c-reactive protein, coronary heart disease, heart failure, Alzheimer’s disease, type 2 diabetes and inflammatory bowel disease.

## Introduction

Circulating concentrations of cholesterol-containing lipoproteins have been linked to risk of atherosclerotic cardiovascular disease (CVD)^1^, in particular coronary heart disease (CHD). Certain circulating lipids have also been implicated in other disorders such as dementia^2^, type 2 diabetes (T2DM)^3^, Crohn’s disease (CD)^4^, rheumatoid arthritis^5^, and some forms of cancers^6^.

The major blood lipid components, free cholesterol, cholesteryl-esters, and triglycerides are transported by lipoprotein particles. Large lipoprotein particles are triglyceride-rich and encompass chylomicrons derived from dietary fat, and very-low density lipoproteins synthesised in the liver. These particles carry a single apolipoprotein B (Apo-B) on the surface (Apo-B 48 for chylomicrons and Apo-B 100 otherwise), and are progressively depleted of triglycerides, through the action of lipoprotein lipase, becoming smaller, denser, and proportionately richer in cholesterol. Lipoproteins, are involved in the process of transporting cholesterol to peripheral tissues (endogenous transport), and are classified according to density gradient centrifugation as (VLDL) very-low-density-, (IDL) intermediate-density- and (LDL) low-density-lipoproteins. Reverse cholesterol transport, from tissues to liver, is mediated by high-density lipoprotein (HDL) particles that are synthesised and released from the liver in nascent form, and which possess membrane-bound apolipoprotein A1 (Apo-A1).

Evidence from non-randomized (i.e., observational) studies, monogenic disorders (FH)^7^, and randomized trials of LDL-C lowering drugs^8,9^ have convincingly shown that higher concentrations of LDL-C increase CHD risk. While non-randomized studies have provided similar evidence^10,11^ of a CHD association with HDL-C and total triglyceride (TG, the aggregate across all lipoprotein particles) concentrations, the lack of successful drugs targeting these blood lipids casts doubt on their potential causal role in CHD. For example, the protective CHD effect of the recently marketed ANGPTL3-inhibitor evinacumab was attributed to its LDL-C reducing ability, despite evinacumab showing strong TG reducing and HDL-C increasing effects^12^.

**Fig. 1:**
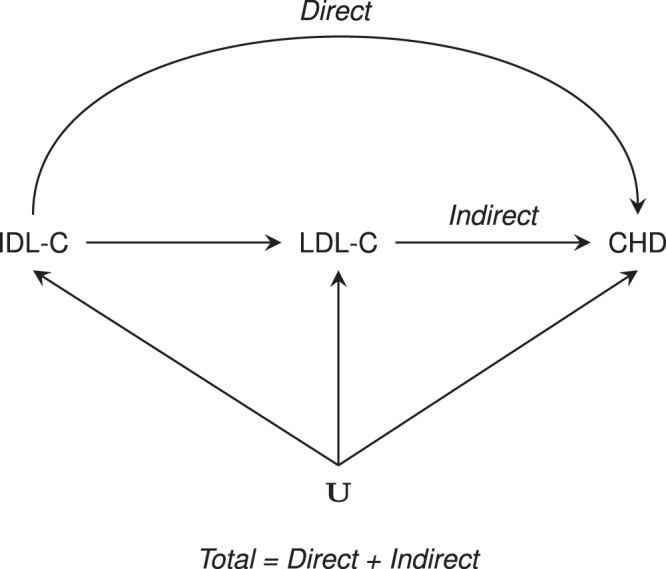
Illustrating the difference between total, direct, and indirect effects, using a hypothetical diagram of intermediate-density lipoprotein cholesterol, low-density lipoprotein cholesterol and coronary heart disease. IDL-C intermediate-density lipoprotein cholesterol, LDL-C low-density lipoprotein cholesterol, CHD coronary heart disease, and common causes (confounders) represented by **U**.

In fact most lipid lowering drugs, including PCSK9 inhibitors, affect lipid fractions beyond LDL-C^8,13,14^. This highlights an inferential challenge, where an exposure may affect disease through multiple independent pathways its (marginal) effect reflects the sum of all pathways and is referred to as the *total effect*. To consider the potentially distinct causal effect of each pathway, mediation analyses can be used to decompose a total effect into multiple, pathway-specific effects; for example into CHD effects attributable to LDL-C, HDL-C and TG (see Fig. 1 for an illustrative example).

Genome-wide association studies (GWAS)^15^ of lipoprotein subfractions quantified by nuclear magnetic resonance (NMR) spectroscopy have identified genetic variants that can be used to undertake Mendelian randomisation (MR) analyses to help ascertain their causal relevance for common disorders. By leveraging genetic variants associated with the exposure(s) of interest, and in the absence of horizontal pleiotropy, MR protects against bias due to confounding^16^ and reverse causation, biases which may befall non-randomized studies. Multivariable MR (MVMR) can additionally account for a genetic variant affecting multiple exposures (e.g., LDL-C as well as HDL-C concentrations), increasing the plausibility of the no-horizontal pleiotropy assumption, as well as identifying the direct effects of the considered exposures^17–19^.

In the current study, we use genetic associations on NMR-measured metabolites and apply two-sample MR to determine the causal relevance of the cholesterol content on different lipoprotein subfractions (including remnant-cholesterol (Rem-Chol), the lipoprotein cholesterol not transported by LDL and HDL), as well as Apo-A1 and Apo-B, on a range of cardiovascular (CVD) outcomes, disease biomarkers, measures of organ or systems function as well as late-in-life non-CVD conditions. MVMR is subsequently performed to ascertain whether causal effects might be independent of the routinely measured blood lipids LDL-C, HDL-C, and TG. We specifically focussed on outcomes with prior evidence of possible lipid involvement including CVD, metabolic disease, inflammatory disease, neurological and oncological disease.

Here, we show that the majority of the considered cholesterol-containing lipoprotein and apolipoproteins affect measures of atherosclerosis, blood pressure, C-reactive protein (CRP), and CHD. We additionally find that a subset of these exposures associate with heart failure (HF), T2DM, Alzheimer’s disease (AD), and inflammatory bowel disease (IBD). MVMR analyses suggest that many of the observed effects act independently of clinically measured lipid fractions: LDL-C, HDL-C and TG.

## Methods

### Available NMR data

To evaluate the consequences of elevated concentration of circulating cholesterol-containing lipoproteins and apolipoproteins, we sourced genetic associations from meta-analyses of Kettunen et al.^15^ and UCLEB^20^ (*n* = 33,029) utilizing NMR-based measurements made using the Nightingale platform on VLDL-C, IDL-C, LDL-C, HDL-C, Rem-Chol, TC, TG, Apo-A1, and Apo-B. Independent replication data on LDL-C, HDL-C, and TG, were available from the Global Lipids genetics Consortium (GLGC^21^, *n* = 188,577) based on clinical chemistry measures. While the UK biobank (UKB) has NMR measurements available for a large sample of participants, it is also a major contributor to the outcome data (see the data availability section). In the presence of sample overlap, weak-instruments may result in anti-conservative behaviour (due to an inflated false positive rate). We therefore used the relatively smaller UCLEB-Kettunen data, which closely follows a two-sample paradigm, where weak-instrument settings do not erroneously inflate the false positive rate^22^.

### Selection of genetic instruments for lipoproteins and apolipoproteins

Genetic instruments were selected from throughout the genome using a F-statistic >24 and a minor allele frequency (MAF) of at least 0.01. Variants were clumped to a linkage disequilibrium (LD) R-squared threshold of 0.10 based on a random sample of 5000 unrelated UKB participants of European ancestry.

Following Schmidt et al. 2020^23^, we repeated the Apo-B and Apo-A1 genome-wide MR analyses, additionally applying a *cis-*MR approach, which is arguably more robust to possible horizontal pleiotropy. For *cis-*MR analysis, variants were selected from within a 50kbp window surrounding *APOB* (ENSG00000084674) and *APOA1* (ENSG00000118137). Given the lower number of candidate instruments in a *cis* region (compared to genome-wide MR) we decreased the F-statistic threshold to 15.

Previous MR studies have often applied a significant *p* value threshold of 5 × 10^−8^ (approximately equal to a F-statistic of 30) to identify instruments with a sufficiently strong exposure association. While this conservative threshold protects against weak-instrument bias, applying a lower F-statistic threshold may beneficially increase the number of available variants and thereby decrease the type 2 error rate. To ensure the results remained sufficiently protected against weak instrument bias, the MR analyses leveraged two distinct exposure GWAS (from UCLEB and GLGC) where the large sample size diminished the influence of potential weak-instrument bias. Additionally, should weak-instrument bias occur the two-sample design prevents erroneous inflation of the false positive rate^22^. Furthermore, we note that in large sample size settings (where the estimated F-statistic approximates the true F-statistic), the multiplicative inverse of the estimated F-statistic approximates the amount of bias^24^: in our case this is between at most 7 and 4% for an F-statistic of 15 and 24, respectively.

### Statistical analyses

Residual LD was modelled through generalised least squares (GLS)^25,26^ implementations of the inverse variance weighted (IVW) and MR-Egger estimators. Here the univariable MR methods provide *total effect* estimates, and multivariable MR (MVMR) implementations of IVW and MR-Egger (both implemented as GLS) were used to estimate *direct effects*, independent from combinations of LDL-C, HDL-C and TG. Additionally, addressing the growing interest in Apo-B as a fundamental cause of atherosclerosis, we explored a MVMR model with Apo-B conditioned on HDL-C and TG, excluding LDL-C due to its high correlation (0.90) with Apo-B (Supplementary Fig. 1).

To minimize the potential influence of horizontal pleiotropy we excluded variants with large leverage or outlier statistics^23,27^ and used the Q-statistic to identify possible remaining violations^27,28^. A model selection framework^28^ was applied to select the most appropriate estimator (IVW or MR-Egger) for each specific exposure–outcome relationship; the Egger correction is unbiased even in the extreme setting where 100% of the selected variants affect disease through horizontal pleiotropy but has markedly less power. The model selection framework (originally developed by Gerta Rücker^29^) utilizes the difference in heterogeneity between the IVW Q-statistic and the Egger Q-statistic, preferring the latter model when the difference is larger than 3.84 (i.e., the 97.5% quantile of a Chi-square distribution with 1 degree of freedom).

Multivariable methods, such as MVMR, may falter when considering (conditionally) multicollinear variables—whose inclusion leads to numerically unstable models with noticeably lower precision^30^, which may result in conditionally weak-instrument settings^31^. For example, the strong correlation between LDL-C and Apo-B (Supplementary Fig. 1) would be anticipated to destabilize a model that includes both. While there are methods specifically designed to address such highly correlated data they assume a complete absence of horizontal pleiotropy, which is unlikely to hold^31,32^ and are computationally prohibitive^31^. We therefore identified and downweighed results likely affected by multicollinearity. Dubious results were identified by gradually extending the MVMR models to first consider the influence of each single covariate (genetic instruments with LDL-C, HDL-C, or TG only), before fitting a *fully conditional* MVMR model including all three blood lipids. After filtering on significance (at an alpha of 0.05), unstable estimates were removed by focussing on exposure-outcome relationships with 60% or higher directional concordance (i.e., significant, and directionally concordant in 3 out of 5 models). The five models constituted estimates of (i) the total effect (from the univariable MR models), and direct effects adjusting for (ii) LDL-C, (iii) HDL-C, or (iv) TG, and (v) all three exposures jointly. When LDL-C, HDL-C, or TG was the exposure of interest, adjustments were made for the two remaining exposures only. After prioritizing the available MR results on significance and model stability (at least 60% directional concordance), we summarized prioritized results using forest plots, and as a network encoding exposure and outcome traits as nodes, with associations represented as arcs. See Supplementary Table 1 for a summary of the methods.

Under the null-hypothesis the *p* values of a group of tests follow an uniform distribution between zero and one^33^. Hence to explore the influence of multiplicity, we evaluated the *overall* null-hypotheses using Kolmogorov-Smirnov (KS)-tests^33^, grouping *p* values by exposure or outcome.

### Software

Analyses were conducted using Python v3.7.4 (for GNU Linux), Pandas v0.25, Numpy v1.15^29^, Seaborn v0.11.5, R v4.0.3^34^ (for GNU Linux), ggforesplot^35^, and Cytoscape v3.8.2 (for GNU Linux). Results were presented as mean difference (MD, for continuous traits) or odds ratio (OR, for binary traits) with 95% confidence interval (95%CI) for increasing blood lipid or lipoprotein concentration, scaled to one standard deviation (Supplementary Table 2).

### Institutional review board approval

All GWAS summary statistics were publicly available, with download URLs provided in the data availability section. For all included genetic association studies, all participants provided informed consent and study protocols were approved by their respective local ethical committee. This research has been conducted using the UK Biobank Resource under Application Number 12113.

### Reporting summary

Further information on research design is available in the Nature Portfolio Reporting Summary linked to this article.

## Results

### Phenotypic correlation and correlation between genetic effect estimates

Aside from an inverse correlation of HDL-C and Apo-A1 with TG and VLDL-C blood concentration, the remaining exposures were strongly and positively correlated (Supplementary Fig. 1). The correlation between the genetic effect estimates for these lipid exposures followed a similar pattern as blood concentrations (Supplementary Fig. 1). See the supplementary results.

### Univariable MR: cardiovascular events and risk factors

**Fig. 2:**
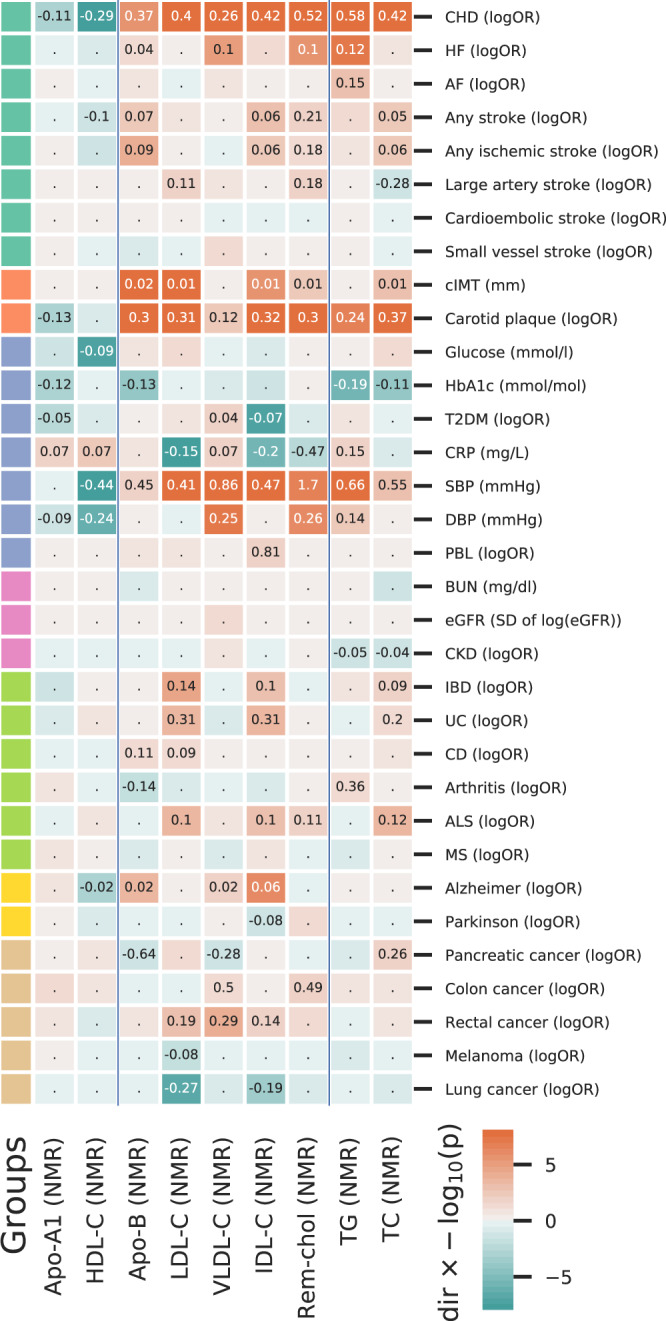
Mendelian randomization estimates of the total effects of a one SD increase in cholesterol-containing lipoprotein and apolipoprotein concentrations. Cells are coloured by the effect direction multiplied by -log_10_(*p* value), with the point estimate (the mean difference or log odds ratio) provided for results with *p* values smaller than 0.05. The *p* values were truncated at 1 × 10^−8^ for display purposes. Analyses are based on a 33,029 subject meta-analysis of Kettunen^15^ and UCLEB^20^. LDL-C low-density lipoprotein cholesterol, HDL-C high-density lipoprotein cholesterol, TG triglycerides, VLDL-C very-low-density lipoprotein cholesterol, IDL-C intermediate-density lipoprotein cholesterol, Rem-chol remnant-cholesterol, TC total cholesterol, Apo-B apolipoprotein-B, Apo-A1 apoliprotein-A1. CHD coronary heart disease, HF heart failure, AF atrial fibrillation, T2DM type 2 diabetes mellitus, CKD chronic kidney disease, IBD inflammatory bowel disease, CD Crohn’s disease, UC ulcerative colitis, ALS amyotrophic lateral sclerosis, MS multiple sclerosis, PBL primary biliary liver cirrhosis, DBP and SBP diastolic and systolic blood pressure, CRP c-reactive protein, HbA1c glycated haemoglobin, BUN blood urea nitrogen, eGFR estimated glomerular filtration rate, cIMT carotid artery intima media thickness.

Higher concentrations of LDL-C, TC, TG, VLDL-C, IDL-C, and Rem-Chol, were associated with higher CHD risk (OR range: 1.29 to 1.79 per SD), while higher HDL-C concentration decreased CHD risk; OR 0.75 (95%CI 0.70; 0.80). HF risk increased with higher concentrations of TG, OR 1.12 (95%CI 1.08; 1.17), VLDL-C, 1.10 (95%CI 1.06; 1.15) and Rem-Chol, OR 1.11 (95%CI 1.06; 1.16); see Fig. 2. Elevated cholesterol-containing lipoproteins were associated with imaging measures of carotid artery atherosclerosis (cIMT and carotid plaque), as well as with SBP and DBP.

### Univariable MR: metabolic events and risk factors

Higher concentration of VLDL-C was associated with increased T2DM risk (OR 1.04 95%CI 1.01; 1.08), while higher IDL-C decreased the risk of T2DM (Fig. 2). A one SD higher LDL-C, IDL-C, and Rem-Chol concentration was associated with lower CRP concentration, while higher HDL-C, TG, and VLDL-C were associated with higher CRP concentration.

### Univariable MR: inflammatory and neurological events

Higher LDL-C concentration was associated with increased the risk of inflammatory bowel disease (OR 1.15 95%CI 1.07; 1.22), ulcerative colitis (UC, OR 1.37 95%CI 1.15; 1.63), and CD (OR 1.10 95%CI 1.00; 1.20). Higher IDL-C and TC had a similar risk increasing effect on IBD and UC. A one SD higher HDL-C decreased Alzheimer’s disease risk (OR 0.98, 95%CI 0.97; 0.99), while AD risk increased with higher concentrations of VLDL-C (OR 1.02, 95%CI 1.00; 1.03) and ILD-C (OR 1.06, 95%CI 1.04; 1.08). Please see the supplementary results and Supplementary Fig. 2 for independent replication of the univariable (*total effects*) for LDL-C, HDL-C, and TG concentration.

### Univariable MR: Apo-B and Apo-A1 concentrations

Higher Apo-B concentration was positively associated with the risk of CHD, (ischaemic) stroke, CD, AD, and with cIMT, carotid plaque and SBP. Conversely, increased Apo-B concentration was associated with lower HbA1c concentration as well as with pancreatic cancer and arthritis risk (Fig. 2). Higher ApoA-1 concentration decreased the risk of CHD, T2DM, carotid plaque, and DBP, while increasing CRP concentrations (Fig. 2). Please see the Supplementary results and Supplementary Fig. 2 for a technical replication using *cis* instruments for Apo-A1 and Apo-B.

### Multivariable MR: to identify effects independent of LDL-C, HDL-C and TG

We applied multivariable MR (MVMR) to investigate whether the above-described causal effect acted independent of the more commonly measured lipids LDL-C, HDL-C, and TG (Supplementary Figs. 3–6).

**Fig. 3:**
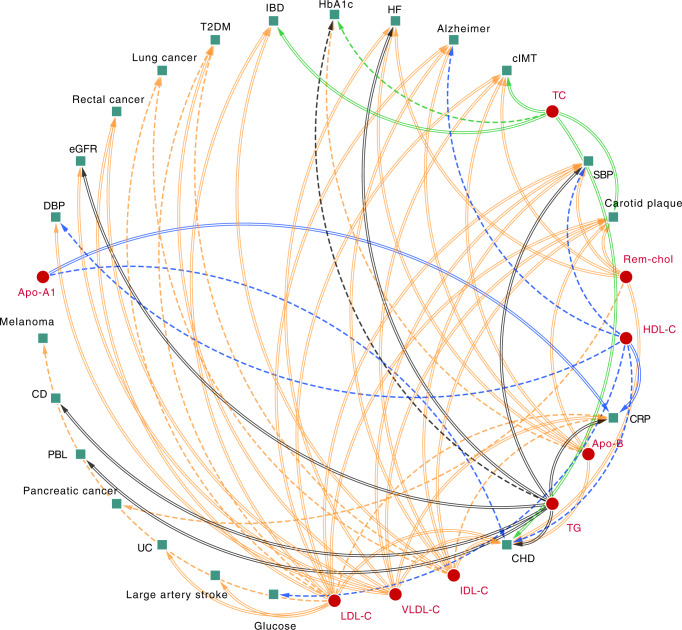
A causal network of phenotypic consequences of higher cholesterol-containing lipoprotein and apolipoprotein blood concentration. The network represents highly supported pathways that likely act independently of LDL-C, HDL-C and TG (which are included as reference). Arcs belonging to the endogenous pathway (VLDL-C, IDL-C, LDL-C, and Apo-B) were coloured *yellow*, arcs for HDL-C and Apo-A1, belonging to the reverse cholesterol transport pathway were depicted in *blue*, TC and TG arcs were represented as black and green, respectively. An increasing effect of a higher exposure concentration was mapped to a double lined arc, a decreasing effect to a dashed arc. An arc was included when the MR effects were significant at an alpha of 0.05 and showed directionally concordant results in at least three of out five potential models (four for LDL-C, HDL-C, and TG): I) total effects, the direct effects conditional II) on LDL-C, III) on HDL-C, IV) on TG, and V) all three blood lipids; see “Methods”. Please see the Fig. 2 for a definition of the abbreviations.

**Fig. 4:**
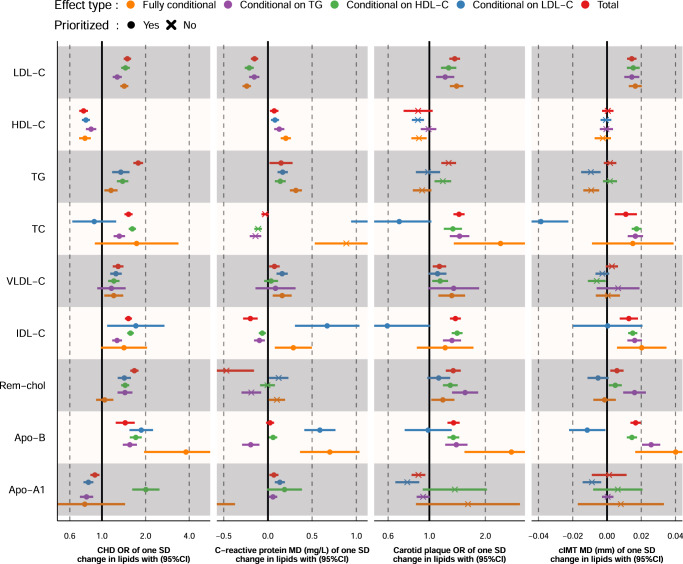
Mendelian randomization effect estimates of a standard deviation change in cholesterol-containing lipoprotein or apolipoprotein concentration on coronary heart disease (CHD), c-reactive protein (CRP), carotid intima media thickness (cIMT), and carotid plaque. Prioritized results reflect associations depicted in the causal network of Fig. 3, where 3 out of 5 (or 4 for LDL-C, HDL-C, and TG) estimates were significant at an alpha of 0.05 and directionally concordant. Total: the total lipid effect, Conditional effects either, represent the blood lipid effect of LDL-C, HDL-C or TG singularly, or off all three blood lipids in a single multivariable MR (fully adjusted) model. Fully adjusted models for LDL-C, HDL-C, or TG exposures only conditioned on two of the three blood lipids (e.g., the fully conditional model for LDL-C exposure only conditioned on HDL-C and TG). Analyses were based on a 33,029 subject meta-analysis of Kettunen^15^ and UCLEB^20^. Estimates are provided as odds ratio (OR) or mean difference (MD) with 95% confidence intervals (95%CI).

**Fig. 5:**
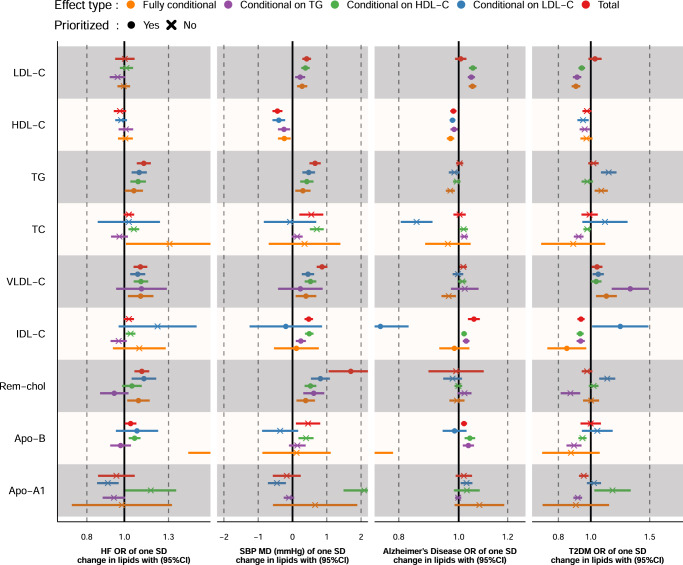
Mendelian randomization effect estimates of a standard deviation change in cholesterol-containing lipoprotein or apolipoprotein concentration on heart failure (HF), systolic blood pressure (SBP), Alzheimer’s disease (AD), and type 2 diabetes (T2DM). Prioritized results reflect associations depicted in the causal network of Fig. 3, where 3 out of 5 (or 4 for LDL-C, HDL-C, and TG) estimates were significant at an alpha of 0.05 and directionally concordant. Total: the total lipid effect, Conditional effects either, represent the blood lipid effect of LDL-C, HDL-C or TG singularly, or off all three blood lipids in a single multivariable MR (fully adjusted) model. Fully adjusted models for LDL-C, HDL-C, or TG exposures only conditioned on two of the three blood lipids (e.g., the fully conditional model for LDL-C exposure only conditioned on HDL-C and TG). Analyses were based on a 33,029 subject meta-analysis of Kettunen^15^ and UCLEB^20^. Estimates are provided as odds ratio (OR) or mean difference (MD) with 95% confidence intervals (95%CI).

MVMR results were ranked based on the number of times a lipid subfraction appeared to affect an outcome (based on the in the “Methods” described prioritization strategy), which is reflected in Fig. 3 as the number of ingoing arcs: CHD, CRP, SBP, carotid plaque, cIMT, HF, AD, T2DM, HbA1c, IBD, lung cancer, rectal cancer, estimated glomerular filtration rate (eGFR), and DBP. The 8 most frequently associated outcomes were presented in Figs. 4 and 5, with all of the MVMR results provided as Supplementary Data 1–12. MVMR results were typically comparable to the univariable analyses, with HDL-C and Apo-A1 decreasing CHD risk, and the remaining lipid exposures increasing CHD risk (Fig. 4). HF risk increased with higher concentrations of VLDL-C OR 1.10 (95%CI 1.02; 1.19), Rem-Chol, Apo-B and TG OR 1.06 (95%CI 1.00; 1.12) (Figs. 3 and 5). AD risk was associated with higher concentration of LDL-C, IDL-C, and Apo-B, while higher HDL-C decreased AD risk: OR 0.97 (95%CI 0.96; 0.98). We also found evidence to support an independent role for VLDL-C increasing T2DM risk OR 1.11 (95%CI 1.04; 1.20), while higher LDL-C (OR 0.90 95%CI 0.88; 0.93) and IDL-C (OR 0.85 95%CI 0.74; 0.97) decreased T2DM risk. We found ubiquitous effects of cholesterol containing lipoproteins and apolipoproteins on CRP, cIMT, carotid plaque, and SBP (Figs. 4, 5).

### Assessing the overall null-hypothesis

**Fig. 6:**
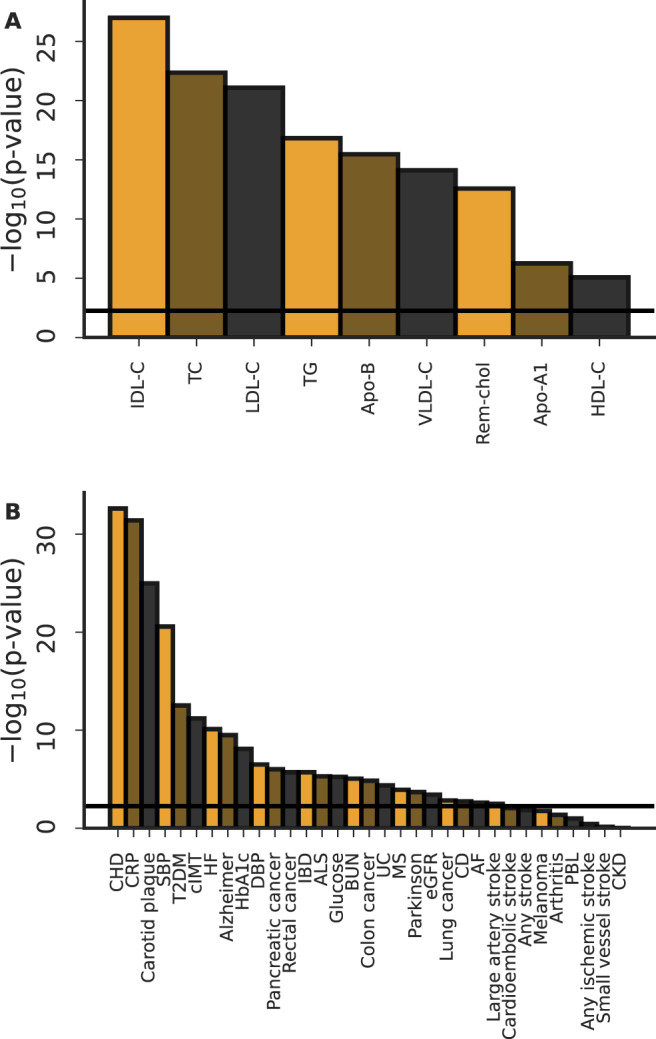
Kolmogorov-Smirnov overall-null hypothesis tests. Kolmogorov-Smirnov goodness-off-fit tests were used to compare an empirical *p* value distribution against the continuous uniform *p* value distribution expected when the strict null-hypothesis holds. **A** Here we grouped the empirical *p* values by exposure and explored whether their distribution agreed with the expected p value distribution when all test would be false-positive. **B** Here we grouped the empirical *p* values by outcome and explored whether their distribution agreed with the expected *p* value distribution when all test would be false-positive. The horizontal lines represent the multiplicity corrected *p* value threshold, dividing an alpha of 0.05 by the number of exposures or outcomes (the number of bars).

To assess to what extent the described results were driven by multiple testing we use Kolmogorov-Smirnov tests (KS-tests) comparing the empirical *p* values distributions against a uniform distribution^33^ (Fig. 6), suggesting results were robust to multiple testing.

## Discussion

We used Mendelian randomization (MR) to catalogue, and prioritize, the biomedical consequences of elevated concentrations of cholesterol-containing lipoproteins beyond LDL-C, HDL-C, and total triglycerides (TG), including remnant cholesterol, IDL-C and VLDL-C, as well as apolipoproteins A1 and B. Findings include that CHD is affected by all of the major cholesterol-rich lipoproteins including HDL-C, IDL-C, VLDL-C, Rem-Chol as well as apolipoproteins A1 and B, and TG, with similar ubiquitous effects observed for cIMT, carotid plaque, and blood pressure. Additionally, we found strong evidence linking higher concentrations of TG, VLDL-C, Apo-B, and Rem-Chol to increased HF risk. Cholesterol-containing lipoproteins, apolipoproteins, as well triglycerides also affected non-CVD traits such as T2DM, CRP, IBD, and AD. Multivariable MR was used to confirm many of these associations act independently of the three widely measured lipid subfractions: LDL-C, HDL-C, and TG.

There has been considerable debate on higher HDL-C potentially reducing CHD risk. The imprecise (univariable MR) OR estimate of 0.93 per SD (95% CI 0·68;1·26) by Voight et al.^36^ is often cited as definitively proving that HDL-C does not affect CHD risk. We note that our estimate OR 0.75 per SD (95%CI 0.70; 0.80) falls completely within the 95%CI provided by Voight et al. Hence our results, suggesting a protective CHD effect of higher HDL-C concentration, are consistent with previous findings. The major difference here is the added precision, as indicated by the confidence interval width, offered by the available larger sample size data (12 K CHD cases by Voight et al. vs 60 K in the current paper). To contextualise the observed HDL-C association with CHD we have collated results from previous univariable and multivariable MR studies (Supplementary Data 13). We find that while there is some variability in statistical significance, results are identical in effect direction, further supporting the observed protective association between higher HDL-C and CHD. Potential explanations for the observed difference in significance include an increase in sample size of the available HDL-C and CHD GWAS’, and the instrument selection strategies (Supplementary Data 13). For example, Holmes et. al. removed HDL-C variants which associated with TG or LDL-C using an *p* value threshold of 0.01, limiting the analysis to 19 variants. It is worth noting that the Richardson et al. study^37^ is the only MVMR study which did not find a statistically significant HDL-C association, which is also the only study that conditioned on both Apo-A1 and HDL-C. Richardson et al. suggested that the univariable association between HDL-C and CHD (OR 0.80 per SD, 95%CI 0.77; 0.89) was attributable to Apo-B. While the regulation of cholesterol homoeostasis is complex, VLDL-C, IDL-C and LDL-C (which all carry Apo-B) play a major role in the endogenous cholesterol transport pathway, whereas HDL-C and Apo-A1 play a dominant role in reverse cholesterol transport^38^, arguing against a strong link between HDL-C and Apo-B concentrations. Empirically, the concentration of HDL-C is only weakly positively correlated to that of Apo-B (0.10, Supplementary Fig. 1) and strongly correlated to Apo-A1 (0.90, Supplementary Fig. 1). As such it seems unlikely that HDL-C exerts its effect on CHD primarily by decreasing Apo-B. Rather, the lack of association between HDL-C and CHD observed by Richardson et al. after adjustment for Apo-B, is more likely a result of forcing two nearly collinear variables (Apo-A1 and HDL-C) into the same multivariable model—a concern acknowledged by Richardson et al. To illustrate this we conducted a MVMR analysis jointly conditioning HDL-C on Apo-B, replacing the Apo-A1 variable by TG (Supplementary Data 12). This analysis confirmed independent CHD associations for HDL-C (OR per SD 0.80, 95%CI 0.74; 0.86) and Apo-B (OR per SD 1.81, 95%CI 1.64; 1.99), where the comparability between the univariable HDL-C association with CHD (OR per SD 0.75, 95%CI 0.70; 0.80) and the HDL-C estimate conditional on Apo-B and TG implies a lack of mediation by these co-variables.

While the considered cholesterol-containing lipoprotein and apolipoproteins have a predominant cardiac and atherosclerotic fingerprint, we found that specific subfractions affected non-CVD diseases including T2DM, AD, and IBD. The association between higher LDL-C concentration and lower risk of diabetes has been observed previously, an effect also observed in meta-analyses of statin trials^39,40^ which may be mediated by effects on adiposity or intracellular metabolism resulting in increased insulin resistance. In the current analysis we now show that IDL-C and VLDL-C affect T2DM independently of LDL-C. Altered cholesterol metabolism has frequently been implicated as a potential risk factor for Alzheimer’s disease through accumulation of phosphorylated tau and amyloid-beta^41,42^. Our MR results suggest changes in LDL-C, IDL-C, Apo-B and HDL-C might be particularly important for AD, potentially leading to interventional targets. For example, the CETP-inhibitor Obicetrapib, which is known to affect the aforementioned lipids, is currently being tested for AD. Cholesterol metabolism is known to interact with inflammatory pathways (marked in our analyses by a CRP association) with oxidized lipoproteins such as LDL-C triggering an immune response^43^. This provides a further (potential) avenue demonstrating how altered lipid metabolism may affect AD risk^44^, as well as explaining the observed LDL-C and IDL-C association with IBD.

This study has employed MR to determine two types of effects (1) the total effect which consists of a direct and indirect effect (where both, or either could be zero), and (2) the direct effect accounting for any potential mediation by the routinely measured lipid fractions LDL-C, HDL-C, and TG (Fig. 1). Both the total effects (e.g., presented in Figs. 2, 4 and 5) and direct effects (e.g., presented in Figs. 3–5) are valid causal effects, and the absence of a direct effect should not be interpreted as disqualifying any observed total effect, or vice versa. We had access to two distinct sets of instruments for LDL-C, HDL-C, and TG, the first from GLGC on about 188,000 participants, and a second set from UCLEB (on about 33,000 participants). Separate analyses using instruments from the two datasets resulted in similar MR estimates (Fig. 2, Supplementary Fig. 2), implying that the presented findings were robust against choices of instruments, as well as source data. It is important to highlight that our genetics instruments were selected on F-statistic >24 which protects against weak instrument bias which (due to the two-sample design) is expected to act towards a null-effect. We specifically utilized MVMR to explore to what extent the observed total effect acted independently from the thoroughly studied exposures LDL-C, TG, or HDL-C. Because MVMR performs a conditional analysis it becomes relevant to also consider conditional F-statistics (Supplementary Table 3), which suggest that MVMR models jointly accounting for LDL-C, HDL-C, and TG, were especially vulnerable conditional weak-instruments. Because of this, analyses were conducted in a two-sample setting, and MVMR-Egger was employed to protect against any potential horizontal pleiotropy not captured by MVMR, ensuring any bias would act towards the null, resulting in conservative findings. While this minimizes the false-positive rate, it also implies (even more than usual) that one should not overinterpret non-significant findings as proof of a null-effect^45^.

In conclusion, we have catalogued and prioritized the phenotypic consequences of cholesterol-containing lipoprotein and apolipoprotein blood concentrations, finding that many of these exposures appear to act independently of the commonly measured blood lipids: LDL-C, HDL-C and TG. We found evidence that CHD and related traits, such as cIMT, carotid plaque, CRP, blood pressure, and HF, are causally affected by many lipid fractions typically including LDL-C, HDL-C, VLDL-C, IDL-C, TG, and apolipoproteins B and A1. Our analyses additionally identified certain non-CVD traits that are more exclusively affected by smaller subset of exposures, such as Alzheimer’s disease (HDL-C, LDL-C, IDL-C, Apo-B), IBD (LDL-C, IDL-C), and T2DM (VLDL-C, IDL-C and LDL-C). The observed pleiotropic effects, where multiple blood lipids affect a single trait, suggest a holistic consideration of lipid metabolism perturbation with respect to disease may be beneficial.

## Supplementary information

Description of Additional Supplementary Files

Supplementary Information

Supplementary Data 1

Supplementary Data 2

Supplementary Data 3

Supplementary Data 4

Supplementary Data 5

Supplementary Data 6

Supplementary Data 7

Supplementary Data 8

Supplementary Data 9

Supplementary Data 10

Supplementary Data 11

Supplementary Data 12

Supplementary Data 13

Supplementary Data 14

Supplementary Data 15

Supplementary Data 16

Reporting Summary

## Data Availability

Summary genetic effect estimates for outcomes were extracted from publicly accessible GWAS on glucose and HbA1c, and C-reactive protein all from the UKB (nealelab.is/uk-biobank; removing low confidence variants), as well as blood pressure (systolic and diastolic), available from Evangelou et al.^46^ (https://www.ebi.ac.uk/gwas/publications/30224653). The CKDGen consortium provided GWAS associations on blood urea nitrogen, estimated glomerular filtration rate, and chronic kidney disease^47^ (https://ckdgen.imbi.uni-freiburg.de/). Genetic associations with primary biliary cirrhosis were available from Jostins et al.^48^ (https://www.ebi.ac.uk/gwas/publications/26394269). A meta-analysis of CHARGE^49^ and UCLEB^20^ provided genetic associations with carotid artery intima media thickness and plaque (https://www.ncbi.nlm.nih.gov/projects/gap/cgi-bin/study.cgi?study_id=phs000930.v6.p1; accession phs000930.v6.p1). CHD data were available for 42,335 cases from CardiogramplusC4D^50^ (http://www.cardiogramplusc4d.org/data-downloads/); 40,585 stroke cases (including four subtypes) from MEGASTROKE^51^ (https://www.megastroke.org/index.html); 47,309 heart failure cases from HERMES^52^ (https://www.ebi.ac.uk/gwas/publications/31919418), 60620 atrial fibrillation cases from Nielson et al.^53^ (https://www.ebi.ac.uk/gwas/publications/30061737), 74,124 type 2 diabetes^54^ cases from DIAGRAM (https://diagram-consortium.org/downloads.html), 32,637 cases of inflammatory bowel disease^55^, 5956 cases of Crohn’s disease^56^ and 6,687 cases of ulcerative colitis^57^ from IIBDGC (https://www.ibdgenetics.org/), 29,880 rheumatoid arthritis cases from Okada et al.^58^ (https://www.ebi.ac.uk/gwas/publications/24390342), 14,498 cases of multiple sclerosis^59^ from the IMSG consortium (https://imsgc.net/), 15,156 amyotrophic lateral sclerosis cases from Rheenen et al.^60^ (https://www.ebi.ac.uk/gwas/publications/27455348), 71,880 cases of Alzheimer’s disease from Jansen et al.^61^ (https://ctg.cncr.nl/software/summary_statistics), and 56,306 cases of Parkinson’s disease from Nalls et al.^62^ (https://www.ebi.ac.uk/gwas/publications/31701892). Finally, we sourced data on pancreatic cancer, colon cancer, rectal cancer, lung cancer and melanoma from Rashkin et al.^63^ (https://github.com/Wittelab/pancancer_pleiotropy). The source data underpinning the figures presented in the main text can be accessed here: 10.5522/04/21647210.v1, with the raw genetic data used in these analyses presented in Supplementary Data 14–15 with a separate readme provided as Supplementary Data 16.
