# Supplementary material for: Biomedical consequences of elevated cholesterol-containing lipoproteins and apolipoproteins on cardiovascular and non-cardiovascular outcomes
Source: Commun Med (Lond). 2023 Jan 20;3:9. doi: 10.1038/s43856-022-00234-0 (PMC9859819; doi:10.1038/s43856-022-00234-0)
Supplement: Supplementary file 1 — Description of Additional Supplementary Files [file 43856_2022_234_MOESM1_ESM.pdf]

Description of Additional Supplementary Files

Filename: SupplementaryData1.tsv
Description: (Multivariable) Mendelian randomization effect estimates for one standard deviation change in LDL-C

Filename: SupplementaryData2.tsv
Description: (Multivariable) Mendelian randomization effect estimates for one standard deviation change in HDL-C

Filename: SupplementaryData3.tsv
Description: (Multivariable) Mendelian randomization effect estimates for one standard deviation change in TG

Filename: SupplementaryData4.tsv
Description: (Multivariable) Mendelian randomization effect estimates for one standard deviation change in VLDL-C

Filename: SupplementaryData5.tsv
Description: (Multivariable) Mendelian randomization effect estimates for one standard deviation change in IDL-C

Filename: SupplementaryData6.tsv
Description: (Multivariable) Mendelian randomization effect estimates for one standard deviation change in Rem-chol

Filename: SupplementaryData7.tsv
Description: (Multivariable) Mendelian randomization effect estimates for one standard deviation change in TC

Filename: SupplementaryData8.tsv
Description: (Multivariable) Mendelian randomization effect estimates for one standard deviation change in Apo-B

Filename: SupplementaryData9.tsv
Description: (Multivariable) Mendelian randomization effect estimates for one standard deviation change in Apo-A1

Filename: SupplementaryData10.tsv
Description: *cis* Mendelian randomization effect estimates for one standard deviation change in Apo-B

Filename: SupplementaryData11.tsv
Description: *cis* Mendelian randomization effect estimates for one standard deviation change in Apo-A1

Filename: SupplementaryData12.tsv
Description: Multivariable Mendelian randomization effect estimates for one standard deviation change in HDL-C, TG, or Apo-B

Filename: SupplementaryData13.tsv
Description: Overview of (previous) Mendelian randomization studies estimating the HDL-C effect on CHD

Filename: SupplementaryData14.tsv.gz
Description: The genetic instrument data used in the univariable (total effect) Mendelian randomization analysis

Filename: SupplementaryData15.tsv.gz
Description: The genetic instrument data used in the multivariable (direct effect) Mendelian randomization analysis

Filename: SupplementaryData16.md
Description: Readme for SupplementaryData14.tsv.gz and SupplementaryData15.tsv.gz
