# Supplementary material for: Biomedical consequences of elevated cholesterol-containing lipoproteins and apolipoproteins on cardiovascular and non-cardiovascular outcomes
Source: Commun Med (Lond). 2023 Jan 20;3:9. doi: 10.1038/s43856-022-00234-0 (PMC9859819; doi:10.1038/s43856-022-00234-0)
Supplement: Supplementary file 2 — Supplementary Information [file 43856_2022_234_MOESM2_ESM.pdf]

Biomedical consequences of elevated cholesterol-containing lipoproteins and apolipoproteins on cardiovascular and non-cardiovascular outcomes

Blood lipoproteins and apolipoprotein

A F Schmidt *et. al.*

Contents

1 Figures **3**

2 Tables **9**

3 Supplementary results **12**

Bibliography **14**

1 Figures

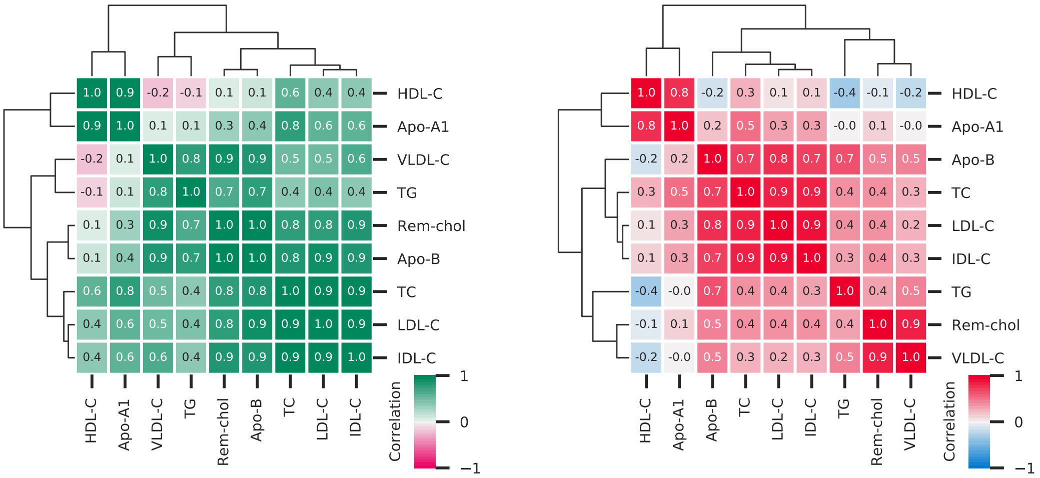

Supplementary Figure 1: Spearman's pairwise correlation between the phenotypic NMR measured blood lipids (*left*: based on a n=14,834 UCLEB[1] sample) and between the genetic association with these blood lipids (*right*: based on a n=33,029 meta-analysis of UCLEB and Kettunen[2]); the margin order was based on hierarchical clustering of the Euclidean distance.

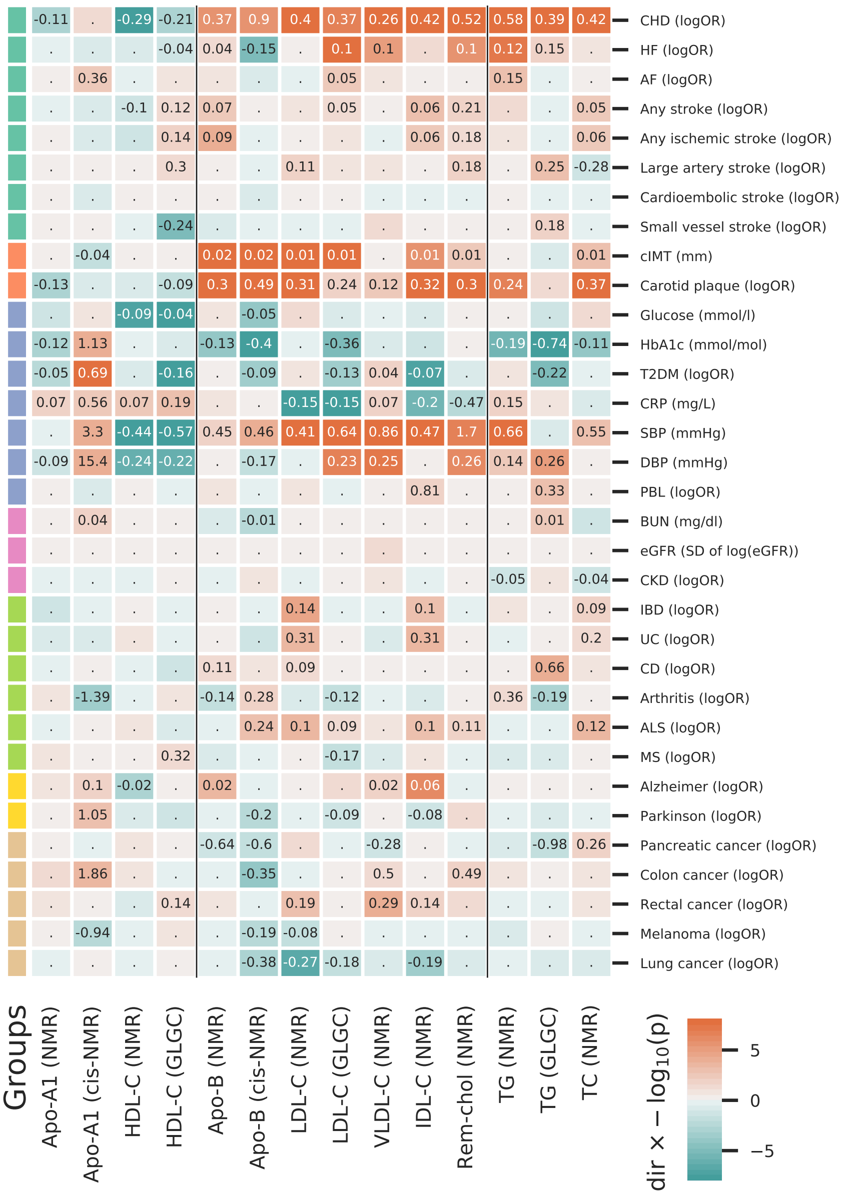

Supplementary Figure 2: Mendelian randomization estimates of the total effects of a one SD increase in cholesterol-containing lipoprotein and apolipoprotein concentration. With independent replication data from the GLGC GWAS [3], and technical replication using *cis* MR analysis of Apo-A1 and Apo-B concentrations.

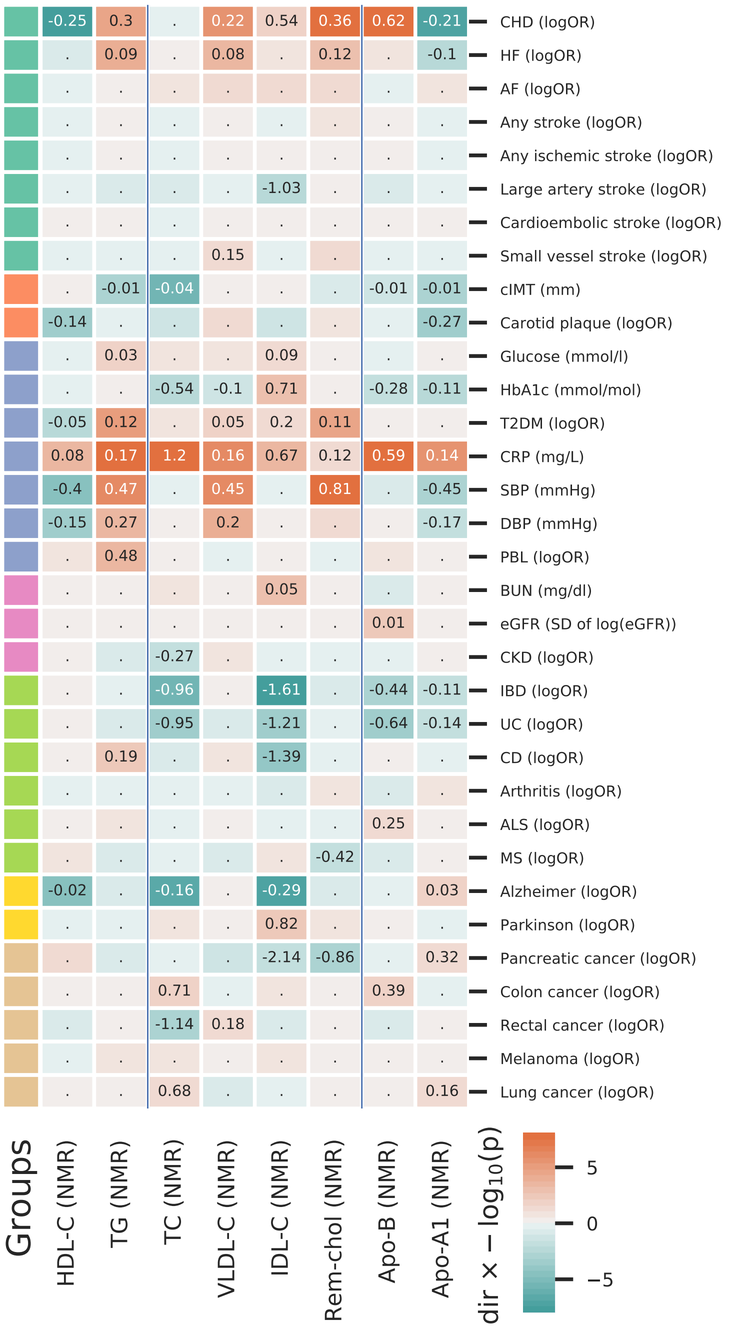

Supplementary Figure 3: Multivariable Mendelian randomization estimates of the direct pathway effect of one SD change in blood lipid, conditional on LDL-C.

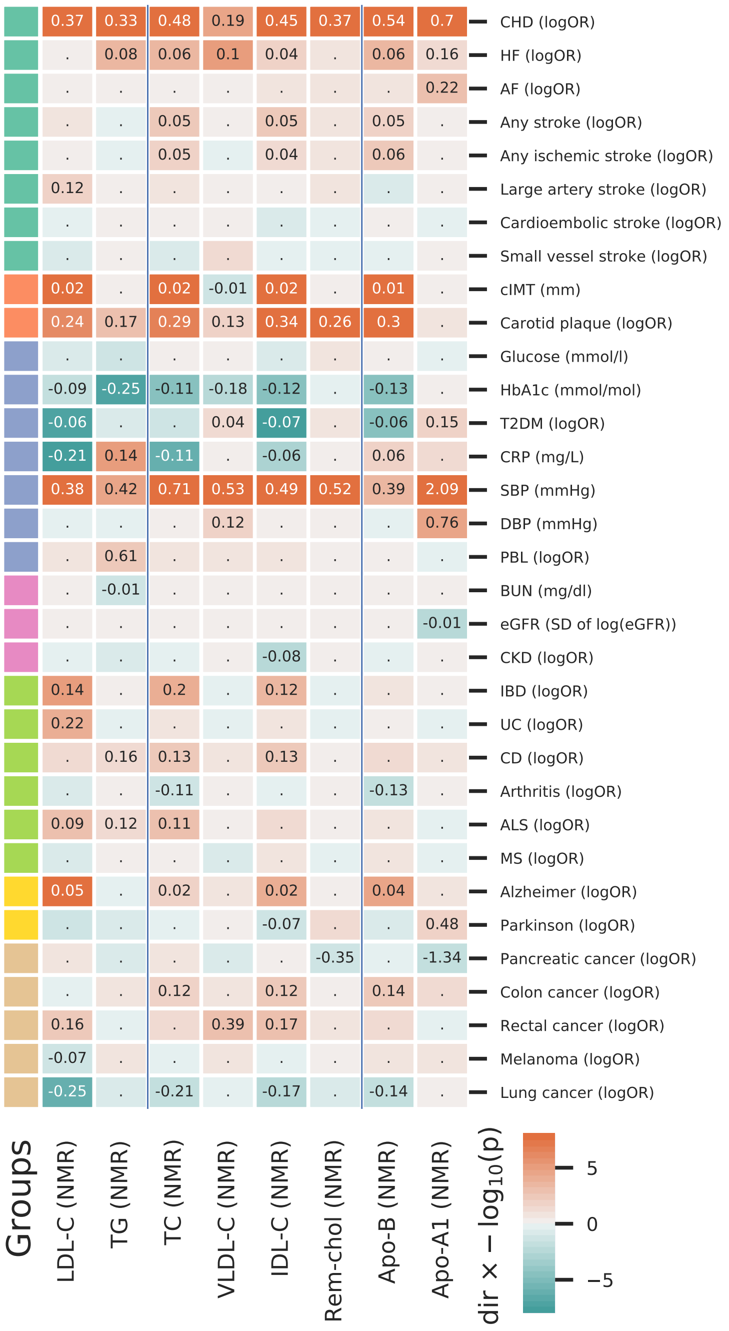

Supplementary Figure 4: Multivariable Mendelian randomization estimates of the direct pathway effect of one SD change in blood lipid, conditional on HDL-C.

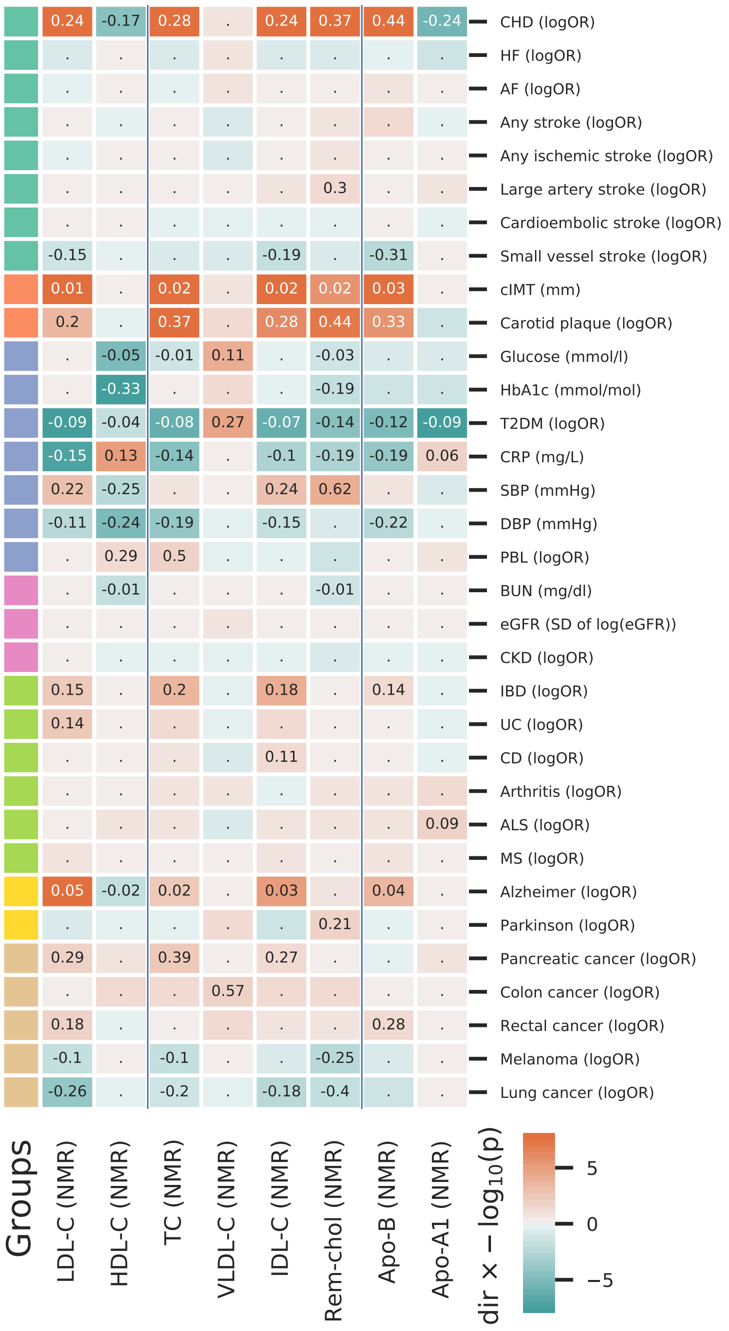

Supplementary Figure 5: Multivariable Mendelian randomization estimates of the direct pathway effect of one SD change in blood lipid, conditional on Triglycerides.

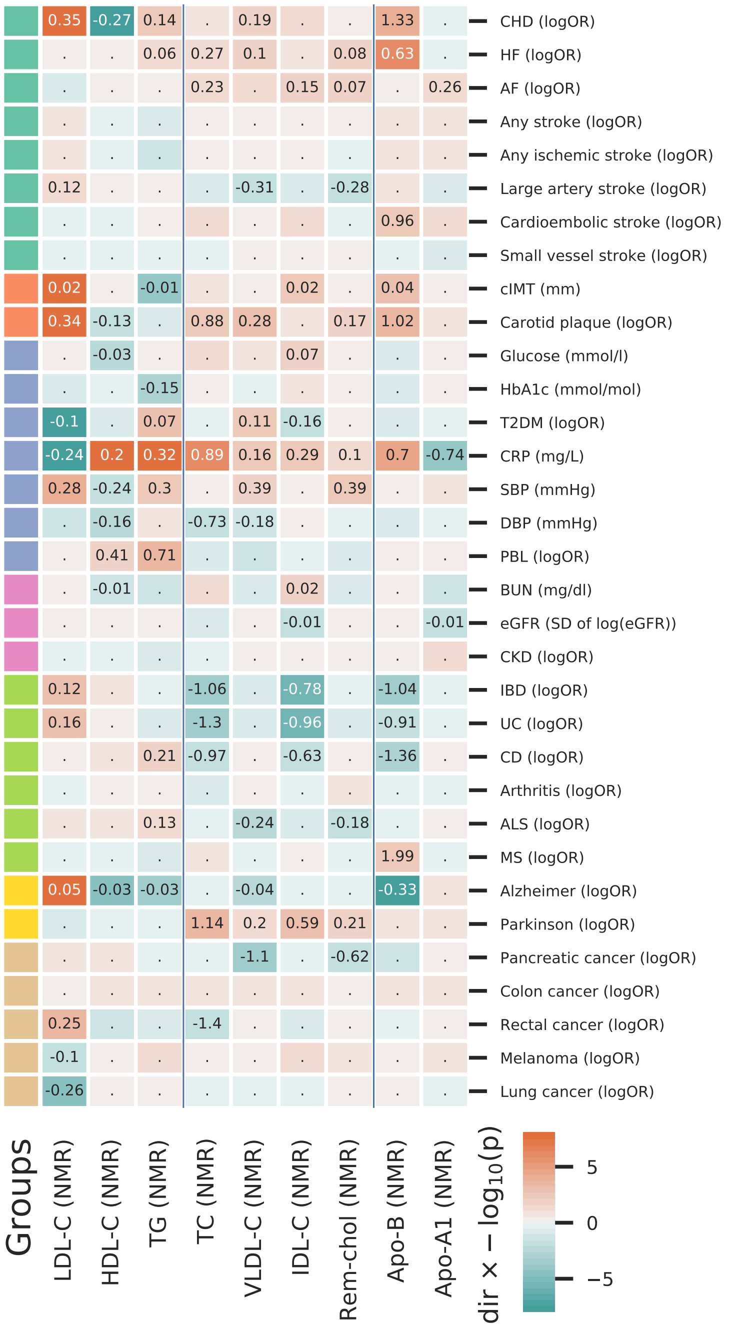

Supplementary Figure 6: Multivariable Mendelian randomization estimates of the direct pathway effect of one SD change in blood lipid, (fully) conditional on LDL-C, HDL-C, and Triglycerides.

2 Tables

Supplementary Table 1: Mendelian randomization analysis synopsis.

**Mendelian randomization steps**
Filter variants on:
• F-statistic larger than 24,<br>• For *cis*-MR:<br>– F-statistic larger than 15,<br>– Within a 50 kbp upstream and downstream window around the gene,<br>• Minor allele frequency of at least 0.01,<br>• Pairwise r-squared less than 0.10.
(MV)MR modelling:
• Inverse variance weighted (IVW),<br>• MR-Egger,<br>• Corrected for residual LD using a UKB reference panel,<br>• Removal of variants with large leverage or heterogeneity statistics,<br>• Rucker framework, selecting the MR-Egger results if $Q_{\text{IVW}} - Q_{\text{Egger}}$ is larger than 3.84.
Prioritization:
• For each exposure and outcome pair:<br>– Determine the *total effect* using univariable MR,<br>– Determine the *direct effects* conditioning on I) LDL-C, II) HDL-C, III) TG, and IV) LDL-C+HDL-C+TG using multivariable MVMR,<br>* Skip duplicate exposure models, e.g., when LDL-C is the exposure of interest do not run an MVMR model including LDL-C twice.<br>– An association between an exposure and outcome pair is considered *prioritized* when it is significant and directionally concordant in at least 60% of the combined univariable and multivariable models.

Supplementary Table 2: The standard deviation of NMR measured blood lipids in mmol/L (total cholesterol, triglycerides, lipoproteins) or g/L (apolipoproteins)

**Blood lipid**	**Standard deviation**
LDL-C	0.646
HDL-C	0.416
TG	0.613
VLDL-C	0.282
IDL-C	0.232
Rem-chol	0.461
Chol	1.238
Apo-B	0.238
Apo-A1	0.276

Supplementary Table 3: Conditional F-statistics for the considered multivariable Mendelian randomiation models

Exposure	Median	Quantile: 0.15	Quantile: 0.85	Conditional model
Apo-A1	15.75	15.62	16.05	LDL-C
HDL-C	18.36	17.84	19.28	LDL-C
Apo-B	11.02	9.11	11.54	LDL-C
VLDL-C	7.88	7.80	8.02	LDL-C
IDL-C	6.41	5.98	6.93	LDL-C
Rem-chol	6.15	6.05	6.46	LDL-C
TG	13.34	12.88	13.79	LDL-C
TC	14.17	12.57	14.82	LDL-C
Apo-A1	15.60	15.52	15.81	HDL-C
Apo-B	18.24	17.40	19.00	HDL-C
LDL-C	25.85	23.58	26.17	HDL-C
VLDL-C	7.49	7.45	7.60	HDL-C
IDL-C	24.86	24.20	25.51	HDL-C
Rem-chol	7.40	7.40	7.65	HDL-C
TG	12.27	11.90	13.42	HDL-C
TC	29.57	28.92	29.95	HDL-C
Apo-A1	16.45	16.26	16.78	TG
HDL-C	14.54	14.37	14.86	TG
Apo-B	18.11	16.85	19.30	TG
LDL-C	19.85	16.94	20.80	TG
VLDL-C	3.13	3.07	3.13	TG
IDL-C	18.96	16.92	19.54	TG
Rem-chol	6.61	6.58	6.75	TG
TC	18.12	16.67	18.64	TG
Apo-A1	3.16	3.01	3.24	LDL-C, HDL-C & TG
HDL-C	11.11	10.94	11.31	LDL-C & TG
Apo-B	2.65	2.61	2.75	LDL-C, HDL-C & TG
LDL-C	16.71	14.95	17.83	HDL-C & TG
VLDL-C	2.74	2.72	2.83	LDL-C, HDL-C & TG
IDL-C	5.49	5.42	5.74	LDL-C, HDL-C & TG
Rem-chol	3.15	3.13	3.29	LDL-C, HDL-C & TG
TG	8.61	8.46	9.37	LDL-C & HDL-C
TC	2.81	2.79	2.93	LDL-C, HDL-C & TG

3 Supplementary results

NMR GWAS participants characteristics

Genetic instruments for cholesterol-containing lipoprotein and apolipoprotein concentrations were sourced from a meta-analysis of Kettunen et al, and the UCLEB consortium. The former included data from Finnish, Estonian, Dutch and German cohorts, with a median age of 46.9 years, median BMI of 26.35 kg/m^2 and 54% women enrolled, see [2]. UCLEB consisted of UK-based cohorts with a mean age of 58.6 years, mean BMI of 26.4 kg/m^2, and 44% women included [1]. Please see the source publications (referenced in the main manuscript) for the subject characteristics of the GWAS outcome data.

Validation of the LDL-C, HDL-C and TG MR effects on disease incidence and biomarkers

The univariable MR effects of LDL-C, HDL-C, and TG were validated using independent replication data from GLGC based on clinical chemistry measurements (Supplementary Figure 2), showing strong agreement both in terms of effect direction and significance.

Univariable MR of Apo-B and Apo-A1 concentration

The MR analyses described for lipid fractions used instruments selected from across the genome (genome-wide MR). In these analyses, horizontal pleiotropy was addressed analytically (see methods). As described by Schmidt *et al.* 2020 [4] MR analyses of protein concentration, such as Apo-B and Apo-A1, may be protected further against horizontal pleiotropy by selecting instrument from a cis window around the protein encoding gene. Here we compared results from genome-wide MR to that of cis-MR and explored agreement (Supplementary Figure 2, and Supplementary Data 8-11).

Higher Apo-B concentration was positively associated with the risk of CHD, (ischemic) stroke, CD, Alzheimer's disease, and furthermore increased cIMT, carotid plaque and SBP. Conversely, higher genetically instrumented Apo-B concentration was associated with lower HbA1c concentration as well as with pancreatic cancer risk. Aside from a directionally discordant effect on HF and arthritis, results from both

cis and genome-wide approached agreed in both effect direction and magnitude, providing empirical support for analytical correction of horizontal pleiotropy. For HF and arthritis, we made further comparisons against the LDL-C, IDL-C and VLDL-C (the carriers Apo-B) effects, providing support for the observed genome-wide Apo-B effect where higher concentrations increased HF risk and lowered arthritis risk (Supplementary Figure 2). Due to the modest number of cis variants for Apo-A1 (2 APOA1 variants compared to 8 for APOB; Tables S10-S13) agreement between both analytical methods was limited. Instead, we focussed on the genome-wide MR results, where we expect the larger number of variant leads to better control of horizontal pleiotropy. The genome-wide MR results suggest that higher ApoA-1 concentration decreases the risk of CHD, and T2DM, decreases carotid plaque size, DBP, while increasing CRP concentrations (Supplementary Figure 2).

Bibliography

[1] Tina Shah et al. “Population genomics of cardiometabolic traits: design of the University College London-London School of Hygiene and Tropical Medicine-Edinburgh-Bristol (UCLEB) Consortium.” In: *PloS one* 8 (8 2013), e71345. ISSN: 1932-6203. DOI: `10.1371/journal.pone.0071345`. epublish.

[2] Johannes Kettunen et al. “Genome-wide study for circulating metabolites identifies 62 loci and reveals novel systemic effects of LPA.” In: *Nature communications* 7 (Mar. 2016), p. 11122. ISSN: 2041-1723. DOI: `10.1038/ncomms11122`. epublish.

[3] Cristen J Willer et al. “Discovery and refinement of loci associated with lipid levels.” In: *Nature genetics* 45 (11 Nov. 2013), pp. 1274–1283. ISSN: 1546-1718. DOI: `10.1038/ng.2797`.

[4] Amand F. Schmidt et al. “Genetic drug target validation using Mendelian randomisation.” In: *Nature communications* 11 (1 June 2020), p. 3255. ISSN: 2041-1723. DOI: `10.1038/s41467-020-16969-0`. epublish.
