# Supplementary material for: Biomedical consequences of elevated cholesterol-containing lipoproteins and apolipoproteins on cardiovascular and non-cardiovascular outcomes
Source: Commun Med (Lond). 2023 Jan 20;3:9. doi: 10.1038/s43856-022-00234-0 (PMC9859819; doi:10.1038/s43856-022-00234-0)
Supplement: Supplementary file 19 — Reporting Summary [file 43856_2022_234_MOESM19_ESM.pdf]

nature research

Corresponding author(s): Amand Floriaan Schmidt

Last updated by author(s): Dec 7, 2022

Reporting Summary

Nature Research wishes to improve the reproducibility of the work that we publish. This form provides structure for consistency and transparency in reporting. For further information on Nature Research policies, see our Editorial Policies and the Editorial Policy Checklist.

Statistics

For all statistical analyses, confirm that the following items are present in the figure legend, table legend, main text, or Methods section.

n/a	Confirmed	
☐	☒	The exact sample size (n) for each experimental group/condition, given as a discrete number and unit of measurement
☐	☒	A statement on whether measurements were taken from distinct samples or whether the same sample was measured repeatedly
☐	☒	The statistical test(s) used AND whether they are one- or two-sided<br>*Only common tests should be described solely by name; describe more complex techniques in the Methods section.*
☒	☐	A description of all covariates tested
☒	☐	A description of any assumptions or corrections, such as tests of normality and adjustment for multiple comparisons
☐	☒	A full description of the statistical parameters including central tendency (e.g. means) or other basic estimates (e.g. regression coefficient) AND variation (e.g. standard deviation) or associated estimates of uncertainty (e.g. confidence intervals)
☒	☐	For null hypothesis testing, the test statistic (e.g. F, t, r) with confidence intervals, effect sizes, degrees of freedom and P value noted<br>*Give P values as exact values whenever suitable.*
☒	☐	For Bayesian analysis, information on the choice of priors and Markov chain Monte Carlo settings
☒	☐	For hierarchical and complex designs, identification of the appropriate level for tests and full reporting of outcomes
☐	☒	Estimates of effect sizes (e.g. Cohen's d, Pearson's r), indicating how they were calculated

Our web collection on statistics for biologists contains articles on many of the points above.

Software and code

Policy information about availability of computer code

Data collection	Data were collected from publicly available GWAS repositories; no specialist software was used for this.
Data analysis	Analyses were conducted using Python v3.7.4 (for GNU Linux), Pandas v0.25,<br>Numpy v1.15, Seaborn, R v3.6.1 (for GNU Linux), ggplot2, and forestplot.

For manuscripts utilizing custom algorithms or software that are central to the research but not yet described in published literature, software must be made available to editors and reviewers. We strongly encourage code deposition in a community repository (e.g. GitHub). See the Nature Research guidelines for submitting code & software for further information.

Data

Policy information about availability of data

All manuscripts must include a data availability statement. This statement should provide the following information, where applicable:

- Accession codes, unique identifiers, or web links for publicly available datasets
- A list of figures that have associated raw data
- A description of any restrictions on data availability

Summary genetic effect estimates for outcomes were extracted from publicly accessible GWAS on glucose and HbA1c, and C-reactive protein (all from the UKB (nealelab.is/uk-biobank; removing low confidence variants), as well as blood pressure (systolic and diastolic), available from Evangelou et al.42. The CKDGen consortium provided GWAS associations on blood urea nitrogen, estimated glomerular filtration rate, and chronic kidney disease43. Genetic associations with primary biliary cirrhosis were available from Jostins et al44. A meta-analysis of CHARGE45 and UCLEB19 provided genetic associations with carotid artery intima media thickness and plaque. CHD data were available for 42,335 cases from CardiogramplusC4D46; 40,585 stroke cases (including four subtypes) from MEGASTROKE47; 47,309 heart failure cases from HERMES37, 60620 atrial fibrillation cases from Nielson et al48, 74,124 type 2 diabetes49 cases from DIAGRAM, 32,637 cases of inflammatory bowel disease50, 5,956 cases of Crohn's disease51 and 6,687 cases of ulcerative colitis52 from IIBDGC, 29880 rheumatoid arthritis

cases from Okada et al53, 14,498 cases of multiple sclerosis54 from the IMSG consortium, 15156 amyotrophic lateral sclerosis cases from Rheenen et al55, 71,880 cases of Alzheimer's disease from Jansen et al56, and 56,306 cases of Parkinson's disease from Nalls et al.57. Finally, we sourced data on pancreatic cancer, colon cancer, rectal cancer, lung cancer and melanoma from Rashkin et al58.

Field-specific reporting

Please select the one below that is the best fit for your research. If you are not sure, read the appropriate sections before making your selection.

☒ Life sciences ☐ Behavioural & social sciences ☐ Ecological, evolutionary & environmental sciences

For a reference copy of the document with all sections, see nature.com/documents/nr-reporting-summary-flat.pdf

Life sciences study design

All studies must disclose on these points even when the disclosure is negative.

Sample size	We did not perform any sample size calculations.
Data exclusions	To improve computational stability, low MAF (below 0.01) variants were excluded (apriori defined).
Replication	Analyses for LDL-C, HDL-C, and TG were replicated using external/independent data
Randomization	While genes cannot be randomly allocated, they are inherited in a random fashion (following Mendel's laws of inheritance).
Blinding	While subjects cannot be actively blinded from their genotype, most subjects would have been unaware of their genotype. Should they have been aware, the implication (if any) on disease would in most cases be insufficiently clear to result in any marked bias.

Reporting for specific materials, systems and methods

We require information from authors about some types of materials, experimental systems and methods used in many studies. Here, indicate whether each material, system or method listed is relevant to your study. If you are not sure if a list item applies to your research, read the appropriate section before selecting a response.

Materials & experimental systems

n/a	Involved in the study	
☒	☐	Antibodies
☒	☐	Eukaryotic cell lines
☒	☐	Palaeontology and archaeology
☒	☐	Animals and other organisms
☒	☐	Human research participants
☒	☐	Clinical data
☒	☐	Dual use research of concern

Methods

n/a	Involved in the study	
☒	☐	ChIP-seq
☒	☐	Flow cytometry
☒	☐	MRI-based neuroimaging
